# Evaluation of a Dedicated Radiofrequency Carotid PET/MRI Coil

**DOI:** 10.3390/jcm11092569

**Published:** 2022-05-04

**Authors:** Mueez Aizaz, Jochem A. J. van der Pol, Roel Wierts, Hans Zwart, Abe J. van der Werf, Joachim E. Wildberger, Jan A. Bucerius, Rik P. M. Moonen, Marianne Eline Kooi

**Affiliations:** 1 Department of Radiology and Nuclear Medicine, Maastricht University Medical Center+, 6202 AZ Maastricht, The Netherlands; mueez.aizaz@mumc.nl (M.A.); jochem.vander.pol@mumc.nl (J.A.J.v.d.P.); roel.wierts@mumc.nl (R.W.); j.wildberger@mumc.nl (J.E.W.); jan.bucerius@med.uni-goettingen.de (J.A.B.); rik.moonen@mumc.nl (R.P.M.M.); 2 CARIM School for Cardiovascular Diseases, 6229 ER Maastricht, The Netherlands; 3 Machnet B.V, 9301 LK Roden, The Netherlands; hzwart@machnet.nl (H.Z.); avanderwerf@machnet.nl (A.J.v.d.W.); 4 Department of Nuclear Medicine, University Medicine Goettingen, Georg-August-University Goettingen, 37073 Goettingen, Germany

**Keywords:** carotid imaging, flexible MRI coils, PET/MRI, attenuation correction

## Abstract

Carotid radiofrequency coils inside a PET/MRI system can result in PET quantification errors. We compared the performance of a dedicated PET/MRI carotid coil against a coil for MRI-only use. An ^18^F-fluorodeoxyglucose (^18^F-FDG) phantom was scanned without and with an MRI-only coil and with the PET/MRI coil. The decay-corrected normalized activity was compared for the different coil configurations. Eighteen patients were scanned with the three coil configurations. The maximal standardized uptake values (SUV_max_) and signal-to-noise ratios (SNR) were calculated. Repeated measures ANOVA was performed to assess the differences in SUV_max_ and SNR between the coil configurations. In the phantom study, the PET/MRI coil demonstrated a slight decrease (<5%), while the MRI-only coil showed a substantial decrease (up to 10%) in normalized activity at the position of coil elements compared to no dedicated coil configuration. In the patient study, the SUV_max_ values for both no surface coil (3.59 ± 0.15) and PET/MRI coil (3.54 ± 0.15) were significantly higher (*p* = 0.03 and *p* = 0.04, respectively) as compared to the MRI-only coil (3.28 ± 0.16). No significant difference was observed between PET/MRI and no surface coil (*p* = 1.0). The SNR values for both PET/MRI (7.31 ± 0.44) and MRI-only (7.62 ± 0.42) configurations demonstrated significantly higher (*p* < 0.001) SNR values as compared to the no surface coil (3.78 ± 0.22), while no significant difference was observed in SNR between the PET/MRI and MRI-only coil (*p* = 1.0). This study demonstrated that the PET/MRI coil can be used for PET imaging without requiring attenuation correction while acquiring high-resolution MR images.

## 1. Introduction

Integrated PET/MRI has emerged as an imaging modality with high potential for atherosclerotic plaque imaging. Functional information provided by PET combined with the superior soft-tissue contrast of MRI makes simultaneous PET/MRI well-suited to visualize all the hallmarks of plaque vulnerability in a one-stop-shop approach [1]. Hybrid PET/MRI also brings other benefits, such as a shorter scan time than sequential PET and MRI exams, improved co-registration of PET and MR images due to the simultaneous nature of the scan [2], the opportunity to use MRI motion information for PET motion correction [3,4,5,6,7], and improved patient comfort and overall convenience compared to sequential scans.

Dedicated radiofrequency (RF) MRI coils allow high-resolution MR imaging, for instance, to visualize the various components of a carotid atherosclerotic plaque [8]. However, the presence of an RF coil inside the PET field of view (FOV) introduces PET photon attenuation, which results in PET quantification errors [9]. Consequently, to obtain accurate attenuation-corrected PET images, the presence of an RF coil in the FOV needs to be taken into account. One solution is to use CT images to create PET attenuation maps (µ-maps) of the coil [10]. The shape and position of flexible coils, such as a dedicated carotid coil, can change depending on the patients’ size and movement during the exam. Furthermore, the CT is performed in a different scanner, which introduces further positional differences. Adjusting these CT images to match the position of the flexible carotid coil during the PET exam is complex and is prone to errors [10].

In the current study, we developed and evaluated a dedicated PET-MRI-compatible coil that was designed to reduce PET attenuation by the coil. Ideally, such a coil would be PET-lucent and thus not require attenuation correction. The objective of our study was to compare the performance of the dedicated PET/MRI carotid coil against the original model designed for MRI-only use in a phantom and patient study. A no surface coil (i.e., built-in body coil) configuration was used to establish the ground truth or reference for comparison.

## 2. Materials and Methods

### 2.1. Coil Design

The design of the PET/MRI coil (PACC-SB30, Machnet B.V. Roden, The Netherlands) is based on the design of the MRI-only coil (PACC-ST30, Machnet B.V. Roden, The Netherlands) with some adjustments for improved PET compatibility.

Both coils are bilateral, 4-channel surface coils with two loop elements per side, covering 105 mm in the anterior-posterior direction, with a width of 60 mm. Each channel uses distributed capacitors to decrease the effect of load changes and has one active PIN decouple circuit and two passive decouple circuits. Per side, the channels are transformer interchannel decoupled. Connection to the scanner is performed using the 4-Channel Flex Coil Interface (Siemens Healthineers, Erlangen, Germany).

**Figure 1:**
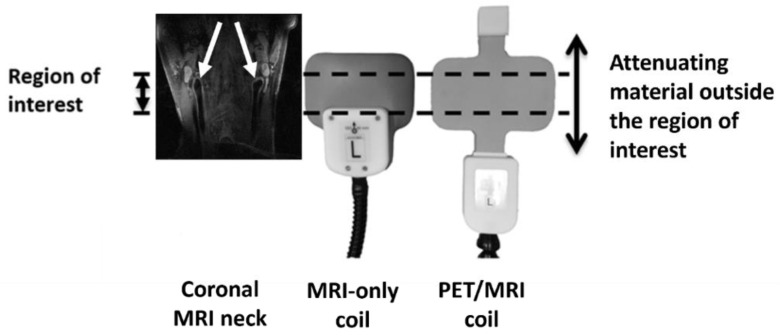
Adaptations to the design of the PET/MRI coil. For the dedicated PET/MRI coil, the electronic housing has been moved further away from the coil elements. The cushioning material that has been used is also thinner and less attenuating. The white arrows point to the left and right carotid arteries.

Based on earlier findings [11,12,13], the highly attenuating polyoxymethylene electronic housing was moved away from the anatomical region of interest. The 0.3 mm thick silver coil conductors were replaced by 35 µm copper coil conductors. The 1.6 mm thick FR4 circuit board (C.I.F.—Circuit Imprimé Français, Buc, France) was replaced by 0.5 mm flexible mylar (RS Components B.V., Haarlem, The Netherlands). The second passive decoupling circuitry was moved to an added remote housing on the opposite side of the coil in the feed-head direction, leaving only four capacitors (one/channel) within the region of interest. Finally, the thickness of the foam padding was reduced from about 26 mm to 15 mm (Figure 1).

### 2.2. Phantom Study

#### 2.2.1. Data Acquisition

A phantom study was set up to determine whether the PET/MRI compatible prototype reduced PET attenuation. A cylindrical phantom, 14 cm in diameter and 20 cm in length, was filled with an aqueous solution of ^18^F-fluorodeoxyglucose (^18^F-FDG; 117.0 MBq). Three measurements were performed: (1) no surface coil (i.e., built-in body coil), (2) with the PET/MRI compatible prototype, and (3) with the original MRI-only coil. Coils were attached to the phantom with a Velcro strap, and the phantom was placed between two foam-rubber holders. Acquisition times were corrected for decay to ensure an equal number of counts per experiment (Table 1). A CT-based µ-map of the phantom without coils was manually registered to the PET emission images to generate attenuation-corrected PET reconstructions, ensuring that any remaining attenuation effect could be attributed solely to the coil. PET reconstruction was performed using the point spread function (PSF) reconstruction algorithm (3 iterations, 21 subsets, matrix size 344 × 344 × 127, voxel size of 2.08 × 2.08 × 2.03 mm^3^, and Gaussian 2 mm filter).

#### 2.2.2. Data Analysis

**Figure 2:**
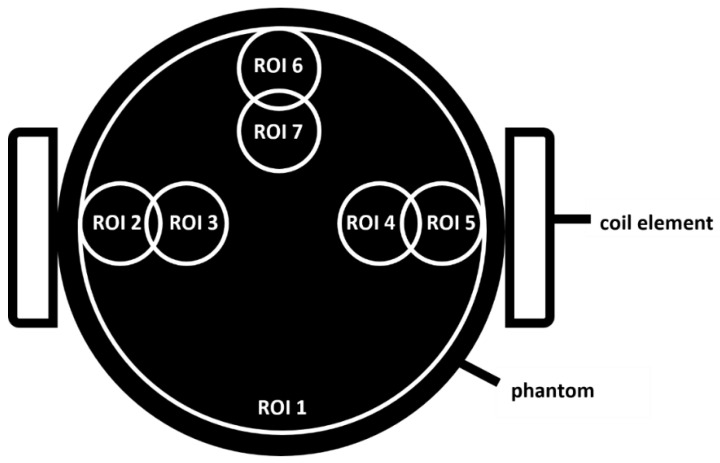
Cylindrical phantom filled with ^18^F-FDG. Seven circular ROIs were drawn. ROIs 2–7 were 2 cm in diameter and positioned 2 and 3.5 cm from the outer edge of the phantom. ROI 1 was 12 cm in diameter and was drawn 1 cm from the outer edge of the phantom and encompasses all other ROIs. All ROIs were extended to slices along the entire length of the phantom. The mean radioactivity concentration (Bq/mL) was calculated for each ROI. This value was then normalized to the mean no surface coil activity in ROI 1.

Seven regions of interest (ROIs) were drawn on the reconstructed images (OsiriX, Pixmeo, Switzerland) as shown in Figure 2. The small ROIs have a diameter of 2.0 cm and were positioned 2.0 and 3.5 cm from the edge of the phantom. The large ROI (ROI 1) contains the entire cross-section of the phantom excluding a 1 cm wide rim. All ROIs were applied to slices along the entire length of the phantom. The mean radioactivity concentration (Bq/mL) was calculated for each ROI. This value was then normalized to the mean no surface coil activity in ROI 1 on a per-slice basis.

To quantify the errors induced by attenuation, the 5-slice (10 mm) average percentage difference from the activity without a surface coil was calculated at three locations: (A) mid-phantom (center of the coil in Z direction), (B) at the MRI-only coil nylon housing (35 mm from center), and (C) at the PET/MRI coil plastic housing (70 mm from center).

### 2.3. Patient Study

#### 2.3.1. Data Acquisition

In this study, eighteen patients scheduled for a regular clinical PET/CT or PET/MRI scan in an oncological setting were included. Institutional medical ethical committee approval was obtained and all patients provided written informed consent. Out of these eighteen patients, three patients aborted the scan before they could be scanned with all three coil configurations. All patients had already received an injection of ^18^F-FDG (mean dose = 213.41 ± 52.45 MBq) for their clinical scan, after which they were scanned for the present study (average duration between injection and start of research scan = 109 ± 19 min). Each patient was scanned in an integrated 3T PET/MRI scanner (Biograph mMR, Siemens Healthineers, Erlangen, Germany) with the three coil configurations (no surface coil, PET/MRI coil, and MRI-only coil). The physiological ^18^F-FDG uptake and clearance could potentially introduce a bias in our results since the three configurations were scanned subsequently. Therefore, the order in which the different configurations were scanned was randomized for each patient. The MRI-based PET µ-maps (Dixon-based automatic tissue segmentation) were acquired without surface coils for all three configurations to ensure potential differences in PET SUVs are due to the presence/absence of the coils only. After positioning the coil, a scout scan was performed to visualize the position of the center of the coil with reference to the carotid bifurcation. Based on the scout scan, the position of the coil was adjusted, if needed, to ensure that the coil center was at the position of the carotid bifurcation before starting the actual scan. For each coil configuration, after the scout scan, a 3-dimensional MR Time-of-Flight (3D TOF) sequence was performed: (repetition time/ echo time (TR/TE) 20 ms/3.6 ms, flip angle (FA) 20°, bandwidth 186 Hz/pixel, acquisition time (TA) 167 s, acquired matrix 256 × 256, reconstructed matrix size 512 × 512, acquired voxel size 0.63 × 0.63 × 1.88 mm^3^, reconstructed voxel size 0.3 × 0.3 × 1 mm^3^). Thereafter, a 3D T1-weighted SPACE sequence (TR/TE 1000 ms/20 ms, echo train length = 49, FA variable, bandwidth 579 Hz/pixel, 2 averages, TA 288s, acquired matrix 192 × 196, reconstructed matrix 384 × 384, acquired voxel size 0.8 × 0.8 × 0.8 mm^3^, reconstructed voxel size 0.4 × 0.4 × 0.8 mm^3^) was acquired. Simultaneously, a 10 min PET scan was performed for each configuration. PET images were reconstructed using the PSF reconstruction algorithm (3 iterations, 21 subsets, matrix size 344 × 344 × 127, voxel size 2.08 × 2.08 × 2.03 mm^3^, and Gaussian 5 mm filter).

#### 2.3.2. Data Analysis

The PET and MR images from each coil configuration from each patient were automatically fused using dedicated software (Syngo.via, version V10B; Siemens) for all 15 patients. Using MR images as reference, ROIs were drawn around the left and right carotid bifurcation, tonsils (2 per patient), salivary glands (2 per patient), and thyroid glands (2 per patient). In one patient, two tumors (squamous cell carcinoma) were present in the field of view, while in another patient, four tumors (Hodgkin’s lymphoma) were detected. In these patients, additional ROIs were drawn encompassing these tumors. In total, 126 ROIs were drawn for each configuration. The maximum standardized uptake value (SUVmax) for each of these regions was recorded. Subsequently, only ROIs that showed considerable tracer uptake (SUV_max_ ≥ 2) were included for further analysis.

**Figure 3:**
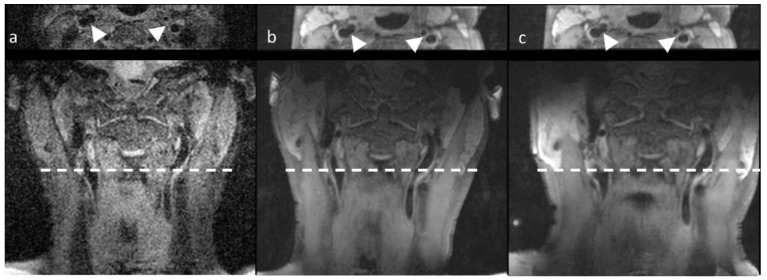
3D T1-weighted SPACE transversal and coronal views acquired with the three configurations of the carotid bifurcation. The upper panel shows transversal images, while the lower panels show coronal images. (**a**) No surface coil, (**b**) MRI-only coil, and (**c**) PET/MRI coil. White arrows in the transversal images indicate the left and right carotid arteries.

To compare MR image quality for the three configurations, signal-to-noise ratios (SNR) were calculated using the 3D T1-weighted SPACE sequence (Figure 3). An ROI was drawn in the sternocleidomastoid muscle at the level of the carotid bifurcation. The mean signal intensity value was used as the signal and the standard deviation as noise (Equation (1)). The same ROIs were used per patient for each configuration after slight adjustments to correct for motion between scans.

(1) $$\;SNR=\left(\frac{mean\;signal\;intensity\;muscle}{standard\;deviation\;muscle}\right)$$

#### 2.3.3. Statistical Analysis

Statistical analyses were performed using SPSS 24.0 (IBM Corporation, Armonk, NY, USA). The SUV_max_ values as well as the SNR values from the patient study were first checked for normality. Repeated-measures ANOVA followed by Bonferroni post hoc test were used to compare the SUV_max_ and SNR values from the three coil configurations. A *p*-value < 0.05 was considered statistically significant.

## 3. Results

### 3.1. Phantom Study

**Figure 4:**
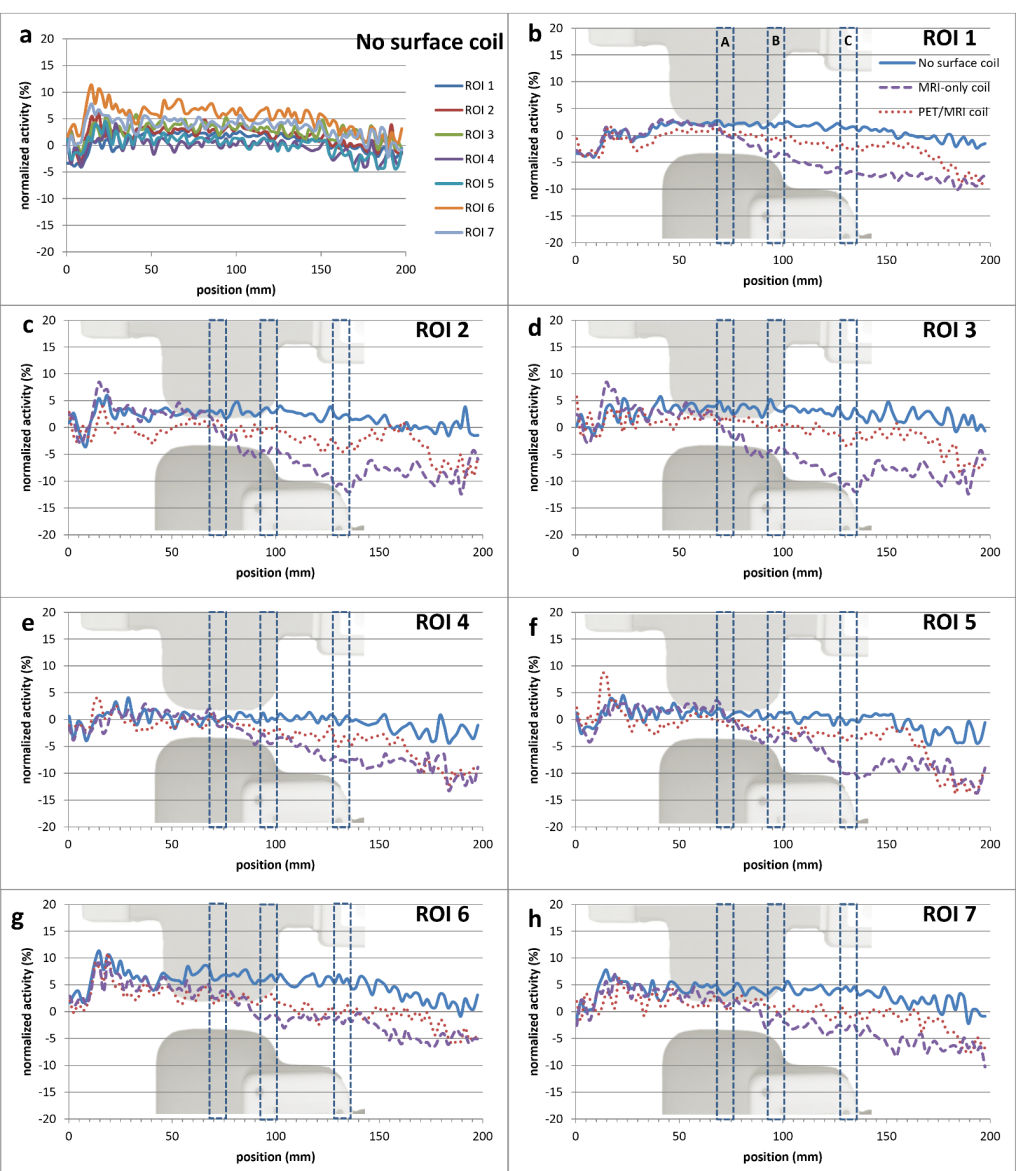
The normalized activity throughout the phantom for each of the ROIs. The approximate position of the PET/MRI coil (top) and MRI-only coil (bottom) can be seen in the background. (**a**) The variation in the no surface coil configuration activity in the seven ROIs. (**b**–**h**) The normalized activities of the no surface coil configuration as well as with the PET/MRI and MRI-only coil. The highest deviation from the no surface coil configuration for each ROI is seen in the region of the electronic housing. In all ROIs the PET/MRI coil shows less deviation from the no surface coil configuration as compared to the MRI-only coil.

Figure 4 shows the normalized activity throughout the phantom for each of the ROIs in the three coil configurations (no surface coil, MRI-only coil, PET/MRI coil). The approximate position of the coil elements is indicated by the images in the background. For no surface coil values, a variability of the measured ^18^F-FDG activity concentration up to 10% was observed between the various ROIs (Figure 4a). In the presence of the PET/MRI or MRI-only coil, deviation from the configuration without a surface coil increases in the regions of the coil elements, with the most severe effects seen near parts containing the electronics’ housing (Figure 4b–h).

The PET/MRI coil showed a lower percentage difference in activity concentration compared to the no surface coil configuration at locations A (center of the phantom) and B (35 mm from the center of the phantom) as compared to the results from the MRI-only coil for all ROIs. At location C (75 mm from the center of the coil), both coils showed similar differences from the no surface coil configuration (Table 2).

### 3.2. Patient Study

**Figure 5:**
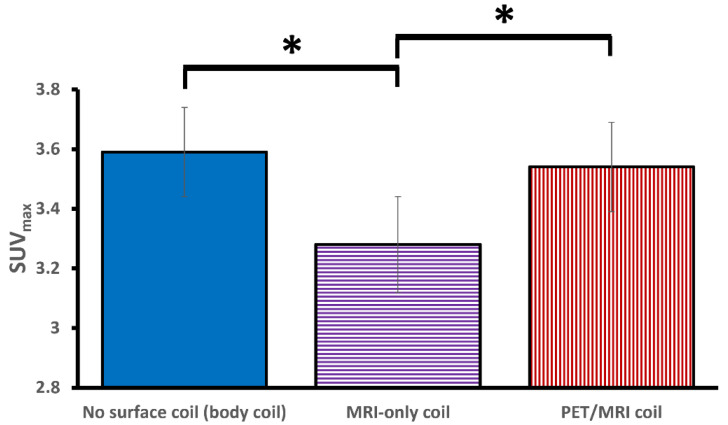
Bar chart showing the mean SUV_max_ values for the three configurations. The error bars represent the standard error. PET/MRI and no surface coil configuration showed significantly higher SUV_max_ values as compared to the MRI-only coil. No significant difference was observed between PET/MRI and no surface coil configuration. The asterisks ("*”) indicate a significant difference in SUV_max_ (*p* < 0.05).

The main characteristics of the patients included in the study are listed in Table 3. After performing the thresholding (SUVmax ≥ 2), 87 values from 15 patients were used in the final analysis. The MRI-only configuration showed significantly lower SUV_max_ values (mean ± standard error (SE)) as compared to the SUV_max_ from the PET/MRI (3.28 ± 0.16 vs. 3.54 ± 0.15, *p* = 0.04) and no surface coil configuration (3.28 ± 0.16 vs. 3.59 ± 0.15, *p* = 0.03) configurations. There was no significant difference between the SUV_max_ values from the PET/MRI and no surface coil (*p* = 1.0) configurations (Figure 5).

**Figure 6:**
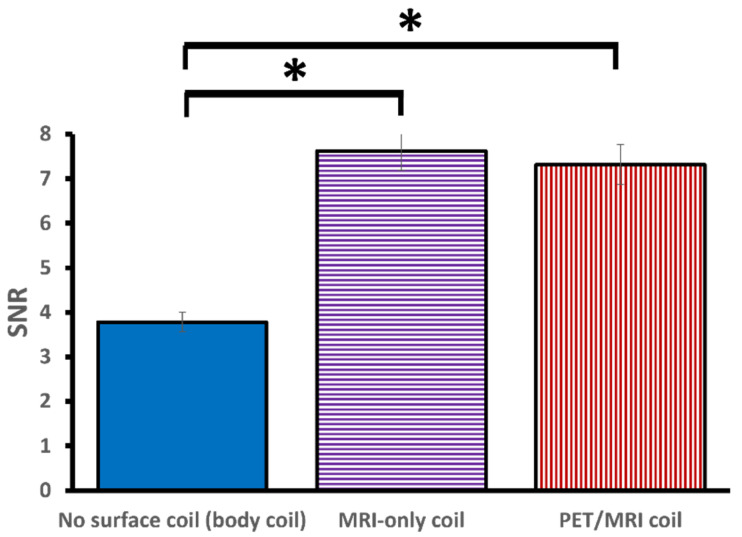
Mean SNR values for the three configurations. The error bars represent the standard error. PET/MRI and MRI-only showed significantly higher SNR values as compared to the configuration without a surface coil. No significant difference in SNR was observed between PET/MRI and MRI-only coils. The asterisks ("*”) indicate a significant difference in SNR (*p* < 0.05).

The overall MRI SNR values (mean ± SE) for PET/MRI (7.31 ± 0.45) and MRI-only (7.62 ± 0.43) were significantly higher as compared to no surface coil configuration (3.78 ± 0.22, *p* < 0.001), while no significant difference was observed in SNR between the PET/MRI and MRI-only coil (*p* = 1.0) (Figure 6).

Comparing the mean SUV_max_ values of the six tumors, the MRI-only coil values were significantly lower as compared to no surface coil (4.88 ± 0.89 vs. 6.28 ± 0.93; *p* = 0.04). No significant difference was present between PET/MRI and no surface coil (5.20 ± 0.43 vs. 6.28 ± 0.93; *p* = 0.43).

## 4. Discussion

This study evaluated the performance of a dedicated bilateral carotid radiofrequency PET/MRI coil that was designed to minimize PET attenuation. More specifically, the attenuation properties as well as the MR image quality were compared with the original four-channel bilateral carotid surface coil used in standard MRI-only scanners, on which the design of the new coil was based. No significant difference was observed in the measured ^18^F-FDG activity concentration (phantom study) and SUV_max_ (patient study) values between the no surface coil and PET/MRI configuration. The MRI-only configuration led to an underestimation of SUV_max_ values (i.e., more attenuation) in the phantom and patient study, respectively, as compared to the values when no surface coil was present. In the phantom study, only in regions further away (75 mm) from the coil center and hence further away from the region most relevant for carotid MRI examinations, deviations in SUV_max_ for the PET/MRI coil showed similar values to those of the MRI-only coil. A significant difference was observed in the SUV_max_ values of the six tumors between the no surface coil and the MRI-only coil. No other combination showed a significant difference in the tumor SUV_max_ values. The MR images that were acquired with the two coils (PET/MRI and MRI-only) showed no significant difference in image quality (SNR).

In the phantom study, we found the average deviation for the MRI-only coil to be 7.3% from the no surface coil configuration for ROI 1. In line with our findings, Eldib et al. [11] performed a similar phantom study with the same MRI-only coil and found a deviation from the no surface coil to be 6.3%. They also concluded that if used without attenuation correction, the MRI-only coil results in significant PET quantification errors. Similarly, other studies [14,15,16] have also shown for different flexible and rigid MR coils that attenuation correction is required for accurate PET quantification.

The non-significant difference in SUV_max_ values between the no surface coil and the PET/MRI coil configuration signifies that attenuation due to the PET/MRI coil is negligible in the region of interest. Previously proposed methods for the attenuation correction of flexible carotid coils were based on adjusted CT-based µ-maps either using MRI and CT visible markers placed on the coil for co-registration [17] using an ultra-short echo time (UTE) acquisition to match the position of the coil on the patient [11,16] or utilizing coil position information derived from a camera setup [18]. The position information extracted from these methods is used to adjust the shape of the CT-based µ-map using non-rigid transformations to match the shape and position of the coil during the MRI exam [17]. However, this method requires complex algorithms to transform the CT-based µ-map, which increases reconstruction time and can introduce errors [10,17].

Apart from the possibility of error introduction due to non-rigid transformations, these methods also do not take into account changes in coil position due to patient movement during the scan. As a consequence of the “PET-lucent” behavior of the newly developed PET/MRI coil, it bypasses the complex CT-based µ-map registration step and can be used inside the PET FOV without requiring further attenuation correction.

As expected, both the MRI-only as well as the PET/MRI coil resulted in a large improvement in SNR. Although the mean SNR for the MRI-only coil is slightly higher than the PET/MRI coil, the individual paired values do not show a pattern of one coil performing better than the other. The high *p*-value (*p* = 1.0) from the SNR comparison between the two coils indicates that the design changes made to reduce attenuation did not affect the MR image quality.

Based on the PET hardware, the PET FoV of the scanner that was used in the present study was 25.8 cm in the feet–head direction. Moving the highly attenuating materials, such as the electronic housing and its components, completely out of the PET FoV would result in a reduction in SNR for MR images due to the large distance between the coils and the electronic components. Instead, while designing the coil, attention was paid to keeping highly attenuating components away from the anatomical region of interest, i.e., carotid bifurcation, while not compromising the SNR. The vast majority of carotid plaques develop around the carotid bifurcation. The length of the plaque in the feet–head direction is in our experience always less than 3 cm [19]. With the carotid bifurcation at the center of the coil, this 3 cm region already overlaps with the electronic housing for the MRI-only coil. For the PET/MRI coil, the housing was moved 5.5 cm from the center of the coil, allowing the uninterrupted passage of the vast majority of photons from the carotid plaque. Our phantom and patient study show that the PET signal can be reliably quantified with these design changes and that the SNR is not compromised.

## 5. Conclusions

In this study, we observed significantly lower PET attenuation with the new PET/MRI coil as compared to the standard MRI-only coil in the region of interest for these dedicated carotid coils. Consequently, PET attenuation correction of the coil is no longer required, bypassing the necessity of complicated coil attenuation correction algorithms and allowing reliable quantitative PET analysis. Furthermore, the lack of attenuation correction requirement equates to more robust and less time-consuming PET scans. Dedicated carotid coils are necessary for diagnostic quality MR images especially when minute structures, such as vulnerable plaque components, need to be visualized. Previous dedicated carotid coils were developed to be used for MRI only and thus are not suited for use inside a PET/MRI system. The PET/MRI coil investigated in this study provides the optimal solution by delivering high-resolution MR images and minimizing PET photon attenuation.

## Figures and Tables

**Table 1:** Acquisition times for each of the configurations to correct for radioactive decay between the different phantom experiments.

Experiment	Time Difference from Start Time (s)	Percentage Activity (%)	Acquisition Time (s)
No surface coil	0	100	1200
PET/MRI coil	1620	84.4	1440
MRI-only coil	3540	69.0	1740

**Table 2:** The average percentage difference from the no surface coil configuration was calculated at three locations: (**A**) mid-phantom (center of the coil), (**B**) at the MRI-only coil electronic housing, and (**C**) at the PET/MRI coil electronic housing. The PET/MRI coil showed a lower percentage difference in activity concentration compared to the no surface coil configuration at location A and B as compared to the results from the MRI-only coil for all ROIs. At location C, both coils showed similar differences from the no surface coil configuration.

	Difference (%) from the Same ROI from the No Surface Coil Configuration					
	A (Center of the Phantom)		B (35 mm from Center of the Phantom)		C (75 mm from Center of the Phantom)	
	PET/MRI Coil	MRI-Only Coil	PET/MRI Coil	MRI-Only Coil	PET/MRI Coil	MRI-Only Coil
ROI 1	−2.8	−5.8	−3.7	−8.3	−6.6	−8.0
ROI 2	−3.8	−7.5	−5.4	−12.4	−7.6	−9.8
ROI 3	−3.3	−6.7	−4.2	−9.6	−7.5	−7.4
ROI 4	−2.7	−4.1	−3.8	−7.5	−8.2	−8.1
ROI 5	−2.9	−4.2	−3.3	−10.2	−9.5	−8.4
ROI 6	−4.0	−7.4	−5.0	−6.5	−6.0	−5.6
ROI 7	−4.3	−6.1	−4.7	−7.0	−7.6	−7.7

**Table 3:** Characteristics of the patients included in the study.

Number of patients	15
Average age (years) (mean ± SD)	63 ± 9.1
Gender (Males/Females)	8/7
Average time difference between injection and start of the scan (minutes) (mean ± SD)	109 ± 19
Average dose (MBq) (mean ± SD)	213 ± 52

## Data Availability

For ethical reasons, the raw data that we collected cannot be made publicly available. The study was approved by the Medical Ethics Committee of the Maastricht University Medical Center, Maastricht, The Netherlands, under the condition that access to the data is granted only to (1) members of the research team, (2) the Medical Ethics Committee members that approved this study, and (3) authorized personnel of the Health Care Inspectorate. Hence, participants did not consent to publicly archive their data. However, requests for anonymous data can be sent to Prof. Dr. M.E. Kooi at eline.kooi@mumc.nl.
